# PERK Signaling Controls Myoblast Differentiation by Regulating MicroRNA Networks

**DOI:** 10.3389/fcell.2021.670435

**Published:** 2021-05-28

**Authors:** Ye-Ya Tan, Yin Zhang, Bin Li, Yang-Wen Ou, Shu-Juan Xie, Pei-Pei Chen, Shi-Qiang Mei, Qiao-Juan Huang, Ling-Ling Zheng, Liang-Hu Qu

**Affiliations:** ^1^MOE Key Laboratory of Gene Function and Regulation, State Key Laboratory of Biocontrol, School of Life Sciences, Sun Yat-sen University, Guangzhou, China; ^2^Guangdong Provincial Key Laboratory of Malignant Tumor Epigenetics and Gene Regulation, Research Center of Medicine, Sun Yat-sen Memorial Hospital, Sun Yat-sen University, Guangzhou, China; ^3^Department of Cardiovascular Medicine, Second Affiliated Hospital of Guangzhou, University of Chinese Medicine, Guangzhou, China; ^4^AMI Key Laboratory of Chinese Medicine in Guangzhou, Guangdong Province Hospital of Chinese Medicine, The Second Affiliated Hospital of Guangzhou University of Chinese Medicine, Guangdong Provincial Academy of Chinese Medical Science, Guangzhou, China

**Keywords:** myoblasts, differentiation, PERK signaling, microRNA network, C2C12 (mouse skeletal myoblasts)

## Abstract

The unfolded protein response (UPR) plays important roles in various cells that have a high demand for protein folding, which are involved in the process of cell differentiation and development. Here, we separately knocked down the three sensors of the UPR in myoblasts and found that PERK knockdown led to a marked transformation in myoblasts from a fusiform to a rounded morphology, which suggests that PERK is required for early myoblast differentiation. Interestingly, knocking down PERK induced reprogramming of C2C12 myoblasts into stem-like cells by altering the miRNA networks associated with differentiation and stemness maintenance, and the PERK-ATF4 signaling pathway transactivated muscle differentiation-associated miRNAs in the early stage of myoblast differentiation. Furthermore, we identified Ppp1cc as a direct target gene of miR-128 regulated by the PERK signaling pathway and showed that its repression is critical for a feedback loop that regulates the activity of UPR-associated signaling pathways, leading to cell migration, cell fusion, endoplasmic reticulum expansion, and myotube formation during myoblast differentiation. Subsequently, we found that the RNA-binding protein ARPP21, encoded by the host gene of miR-128-2, antagonized miR-128 activity by competing with it to bind to the 3′ untranslated region (UTR) of Ppp1cc to maintain the balance of the differentiation state. Together, these results reveal the crucial role of PERK signaling in myoblast maintenance and differentiation and identify the mechanism underlying the role of UPR signaling as a major regulator of miRNA networks during early differentiation of myoblasts.

## Introduction

The unfolded protein response (UPR) is an evolutionarily conserved signaling pathway that responds to perturbations in endoplasmic reticulum (ER) homeostasis. The UPR plays crucial roles in physiological and pathological processes that have high requirements for protein folding when certain cell types are subjected to internal or external stress or even under normal development conditions (Hetz et al., 2020). The UPR is known to be mediated by three ER transmembrane sensors, each of which plays an important role in normal development: the RNA-dependent protein kinase-like ER eukaryotic translation initiation factor 2 alpha kinase (PERK), inositol-requiring enzyme 1 (IRE1), and activating transcription factor 6 (ATF6) (Godin et al., 2016; Mitra and Ryoo, 2019). The IRE1 and ATF6 signaling pathways mainly activate UPR downstream genes through the downstream effectors X-box-binding protein 1 (XBP1) and 50-kDa nuclear ATF6 (p50ATF6) to alleviate the ER stress response, but the PERK signaling pathway is dependent on eukaryotic translation initiation factor 2α (eIF2α) phosphorylation, which causes a reduction in global protein synthesis while selectively allowing the translation of mRNAs with specific upstream open reading frames (uORFs) in their 5′ untranslated regions (UTRs) (Haze et al., 1999; Calfon et al., 2002; Pakos-Zebrucka et al., 2016). The IRE1 branch is the primary UPR signaling pathway in mammals and has been previously recognized for its essential requirement in the developing liver and in the B cell lineage (Reimold et al., 2000; Reimold et al., 2001). Vertebrate species encode two isoforms of ATF6 (ATF6α and ATF6β), and experiments in mice with single- or double-gene knockout have demonstrated that ATF6α and ATF6β have a necessary but overlapping function in the early embryonic stage (Yamamoto et al., 2007). It has been recently found that dysfunction of ATF6 impedes mesodermal fate specification during development (Kroeger et al., 2018). The extraordinary role of the PERK branch of the UPR in secretory cells with a high protein load has been widely investigated by making use of gene targeting: PERK-knockout mice show dysfunction of islet β cells, pancreatic acinar cells, and cells within the skeletal system (Harding et al., 2001; Zhang et al., 2002; Iida et al., 2007; Gallot et al., 2019). Aside from the function of PERK in secretory cells, inhibition of PERK signaling results in stem cell accumulation in organoid cultures of the primary intestinal epithelium, which disrupts the differentiation of intestinal epithelial stem cells (Heijmans et al., 2013). More importantly, the regulatory effects of PERK also rely on activating transcription factor 4 (ATF4), which is the main downstream transcription factor of PERK-eIF2α-dependent translation in response to various physiological requirements. Notably, ATF4-knockout mice show developmental defects, including pancreatic hypertrophy and severe skeletal defects (Iida et al., 2007; Wang et al., 2009). The physiological activity of the PERK signaling pathway has been found to be tightly associated with the differentiation of some stem cells and embryonal organs; however, the general mechanism underlying this branch of the UPR in developmental processes remains to be elucidated.

Myogenesis is an elaborate process that involves the maintenance of stem and progenitor cells, lineage specification, and terminal differentiation (Bentzinger et al., 2012). During the early phase of embryonic development, progenitors are specified and determined to be myoblasts, and the first muscle fibers are established with differentiated mononucleated myocytes. Myocytes fuse to form multinucleated myofibers that reach a steady state, and progenitors will enter quiescence and henceforth reside within the matured muscle as satellite cells during the late phase of embryonic development (Tajbakhsh, 2009). Satellite cells have the potential to expand mitotically and differentiate to repair the muscle tissue and reestablish homeostasis, when mature muscle is damaged (Rudnicki et al., 2008). Many underlying signaling mechanisms control the genetic networks that promote myogenesis. Myogenic regulatory factors (MRFs), collectively expressed in the skeletal muscle lineage, are required for the terminal differentiation of myoblasts and the expression of myotube-specific genes. Myocyte enhancer factor 2 (MEF2) is a lynchpin for potentiating the function of MRFs through transcriptional cooperation. Satellite cells, the major mediators of myofiber regeneration in adults, are dominated genetically by transcription factor paired box 7 (PAX7), which further regulates downstream myogenic factors, such as myoblast determination protein 1 (MyoD) (Seale et al., 2000; Punch et al., 2009). *In vitro*, myoblast differentiation serves as a powerful model system for studying key signaling mechanisms that control genetic networks during myogenesis (Braun and Gautel, 2011). Intriguingly, the morphological hallmark of the myotube is a highly developed ER network, suggesting that the myogenic process is accompanied by ER membrane expansion, a physiological process facilitated by the UPR (Pedersen and Febbraio, 2012; Afroze and Kumar, 2019).

MicroRNAs (miRNAs), a new class of cell lineage regulators, are also involved throughout the process of embryonic development and cellular differentiation (Ivey and Srivastava, 2010; Vidigal and Ventura, 2015; Singh et al., 2020). Most importantly, many miRNAs, especially muscle-specific clusters, such as the miR-1a and miR-133 families, are required for muscle development and must be subject to strict dynamic regulation during rapid developmental transitions or changes in the cellular environment (Rao et al., 2006; Liu et al., 2007; Braun and Gautel, 2011). The well-established miR-206 is capable of regulating myogenesis by a negative-feedback mechanism, in which miR-206 is upregulated by MyoD and targets *Pax7* mRNA (Chen et al., 2010). Additionally, the highly coordinated and time-dependent UPR can be reciprocally regulated by flexible miRNA networks, which activate the adaptation program without triggering cell death pathways and induce gene transcription tailored to the cellular demand (Behrman et al., 2011; Amodio et al., 2016; Xu et al., 2016). PERK has been shown to contribute to the regulation of regenerative myogenesis, which is essential for the satellite cell homeostasis and differentiation of activated satellite cells into the myogenic lineage (Zismanov et al., 2016; Xiong et al., 2017). By using a myoblast differentiation system *in vitro*, we attempted to explore the functional requirements of the UPR branches during myogenesis and decipher the mechanism underlying the role of PERK signaling in controlling muscle differentiation by regulating miRNA networks.

In our study, knocking down PERK in C2C12 myoblasts resulted in a significantly changed expression of miRNAs related to pluripotency and differentiation. Then, we found that ATF4 directly mediated the transcription of some myomiRs during the early stage of myoblast differentiation. In addition, we found that PERK-regulated miR-128 targeted protein phosphatase 1 catalytic subunit gamma (*Ppp1cc*) mRNA, which is a key regulator of the UPR signaling pathway. We also found that cyclic AMP-regulated phosphoprotein 21 (ARPP21), the host gene of miR-128-2 encoding an RNA-binding protein, antagonized miR-128 activity during late myogenesis. Based on these data, we propose a model in which a feedback loop between PERK signaling and miR-128 promotes myoblast differentiation.

## Materials and Methods

### Animal

C57/BL6 mice were purchased from the Guangdong Medical Laboratory Animal Center, Guangdong, China. Mice were housed in the animal facility and had free access to water and standard rodent chow. Fetal (E12.5, E15.5, and E18.5) and postnatal (2 and 8 weeks) hind limb muscles were isolated for RNA and protein extraction.

### Cell Cultures and Treatments

The mouse skeletal myoblast cell line C2C12 was obtained from the Shanghai Institute of Cell Biology, Chinese Academy of Science. Cells were cultured in a growth medium (GM), which consists of high-glucose Dulbecco’s modified Eagle’s medium (DMEM, Thermo Fisher) supplemented with 10% FBS (Thermo Fisher) and 1% penicillin/streptomycin (Thermo Fisher), at 37°C in 5% CO_2_. Cells were plated and cultured to 100% confluence and were then transferred to a differentiation medium consisting of DMEM containing 2% horse serum (Thermo Fisher) and 1% penicillin/streptomycin for further culture. To inhibit the activity of the PERK signaling pathway, C2C12 cells were incubated with GSK2606414 (Selleck), and PBS was used as the negative control. Transfection of plasmid DNA was performed using ViaFect^TM^ Transfection Reagent (Promega), and all RNA transfections were performed at a final concentration of 50 nM using Lipofectamine 2000 (Thermo Fisher) according to the manufacturer’s instructions.

### RNA Extraction and qPCR Assays

Total RNA was extracted from mouse muscles or C2C12 cells with TRIzol reagent (Invitrogen) according to the manufacturer’s instructions. First-strand cDNA for PCR analyses was synthesized with a PrimeScript^TM^ RT reagent kit (Takara). Real-time PCR was performed using SYBR Premix ExTaq^TM^ (Takara) in a sequence detection system (Thermo Fisher). The *C*t values were first normalized to those of the endogenous control (GAPDH or U6) and then normalized to those of the control group (ΔΔ*C*T method) to calculate the fold change between the control and experimental groups. All primer sets were synthesized by Synbio Technologies, and all primer sequences are listed in Supplementary Tables 5–7.

### Protein Isolation and Western Blot Analysis

Tissue was homogenized and then lysed in ice-cold RIPA buffer (50mM of Tris–HCl (pH 7.4), 150mM of NaCl, 0.1% SDS, 1% sodium deoxycholate, 1% Triton X-100, 2 mM of EDTA, and 1× protease inhibitor cocktail). Samples were then centrifuged for 20 min at 4°C. Total protein extracts were loaded and separated by SDS-polyacrylamide gel electrophoresis (SDS-PAGE) and were then transferred to nitrocellulose membranes (Whatman). Membranes were then blocked with 5% milk for 1 h. Membranes were incubated with primary antibodies against MYOD (Proteintech Cat# 18943-1-AP), MEF2C (Proteintech Cat# 10056-1-AP), MyHC (R&D Cat# MAB4470), PERK (CST Cat# 3192), p-PERK (CST Cat# 3179), phospho-eIF2α (1:1000, CST Cat# 3398), eIF2α (CST Cat# 9722), ATF4 (CST Cat# 11815), p-ATF4(Ser219) (Thermo Fisher Cat# PA5-105835), ATF6 (CST Cat# 65880), IRE1α(CST Cat# 3294), PPP1CC (Proteintech Cat# 55150-1-AP), ARPP21 (Proteintech Cat# 55150-1-AP), c-Myc (CST Cat# 5605), KLF4 (CST Cat# 4038), Nanog (CST Cat# 8822), SOX2 (CST Cat# 4962), OCT4 (CST Cat# 2840), PAX7 (Proteintech Cat# 20570-1-AP), and GAPDH (Proteintech Cat# 55150-1-AP) overnight at 4°C. Horseradish peroxidase-conjugated secondary antibodies were used to detect the primary antibodies and protein signals were then visualized using Chemiluminescent HRP Substrate (Millipore, WBKLS0500).

### Vector Construction

3′UTRs containing the putative binding site for miR-128 were cloned into the psiCHECK2 vector (Promega) backbone using the Xho I/Not I restriction enzymes. Genomic fragments of the miRNA precursors were cloned into pcDNA6.2 (Invitrogen). The shRNA oligonucleotides were annealed and cloned into the pLKO.1-TRC plasmid with Age I/EcoR I sites. Cignal 45-pathway reporter arrays, the ATF4 pathway reporter, the ATF6 pathway reporter, and the IRE1 pathway reporter, which measure the activity of the corresponding pathways were purchased from Qiagen. The ATF4 and PPP1CC coding sequence (CDS) was cloned in-frame into a reconstructive TRE3G vector with promoter EF1α for stable expression. The ARPP21-coding sequence (CDS) was cloned in-frame into the p3XFLAG-pCGH vector for stable expression. The primers used for vector construction are listed in Supplementary Table 4.

### Lentiviral Transduction for the Establishment of Stable Cell Lines

The lentiviral vectors were co-transfected with the packaging vectors psPAX2 and pMD2.G (Addgene) into 293T cells. Supernatants containing viral particles were harvested and were then filtered through a 0.45-μM filter. To establish stable cell lines, C2C12 cells were infected with lentiviral particles, and polybrene (6 μg/mL, Sigma) was added to facilitate infection. After 48 h, the infected cells were subjected to selection in medium containing puromycin (3 μg/mL, Sigma) for 5 days.

### Small RNA Sequencing and Analysis

Small RNA sequencing was performed by RiboBio Co., Ltd. Briefly, total RNA or purified sRNA fragments of the samples were extracted and were first ligated to the 3′-terminal and 5′-terminal linkers and then reverse-transcribed into cDNA. PCR amplification was carried out, and the gel was then cut to recover the target fragment library. *In silico* sequencing was performed on libraries that passed the quality inspection. Small RNAs were annotated by direct alignment to the genome and to various known RNAs using bowtie. All miRNA target sites were further annotated using a mouse genome assembly (mm10). Functional enrichment analysis was performed using the DAVID functional annotation tools (Huang da et al., 2009).

### Chromatin Immunoprecipitation (ChIP)

Chromatin immunoprecipitation (ChIP) was performed as described previously (Dong et al., 2014). Briefly, cross-linking was performed with C2C12 cells with 1% formaldehyde (Sigma), and nuclei were extracted using cell lysis buffer (20 mM of Tris–HCl (pH 8.0), 85mM of KCl, and 0.5% NP-40). Nuclei were lysed with nuclear lysis buffer [10 mM of Tris–HCl (pH 7.5), 1% NP40, 0.5% deoxycholate, and 0.1% SDS]. Chromatin/DNA complexes were sheared in a sonicator. Sonicated lysates were cleared and incubated overnight at 4°C with magnetic beads coupled to an anti-ATF4 antibody. The precipitated chromatin was eluted and reverse cross-linked in ChIP Elution Buffer (1% SDS and 0.1 M of NaHCO3) containing proteinase K and RNase A for 2 h at 65°C. The DNA was recovered and purified using a Qiagen PCR purification kit. Semiquantitative PCR or qPCR was performed to analyze the immunoprecipitated DNA. Primers are listed in Supplementary Table 8.

### Luciferase Reporter Assays

C2C12 cells were transfected with different kinds of reporter plasmids for 48 h. Cells were then lysed in passive lysis buffer, and luciferase activities were measured with a Dual Luciferase Assay Kit (Promega) according to the manufacturer’s instructions.

### Migration Assays

For the migration assay, C2C12 cells transfected with miR-128 were suspended in 200 μL of FBS-free medium and were then seeded into the upper chamber of transwell inserts (8 μM pore size, Costar). The lower chamber of the transwell inserts was filled with 750 μL of medium supplemented with 10% FBS, which functioned as a chemoattractant. After 24 h of incubation at 37°C, cells that migrated to the lower surface of the insert membrane were fixed with methanol and stained with 0.1% crystal violet.

### Cell Counting Kit-8 (CCK-8) Assays

For CCK-8 assays, cells were seeded at a concentration of 1 × 10^3^ cells/well in 96-well plates. Cell numbers were quantified using CCK-8 reagent (Cat# CK04, DOJinDO) at the indicated time according to the manufacturer’s instructions.

### Colony Formation Assays

For colony formation assays, 1 × 10^3^ cells were seeded into six-well plates. Colonies, which were allowed to form for 5 days after plating, were stained with a crystal violet solution and counted. Assays were done in triplicate.

### Fluorescence Microscopy

C2C12 cells grown on glass coverslips were stained according to the manufacturer’s instructions. The endoplasmic reticulum was stained using l g/mL of Alexa Fluor^®^, 647 Concanavalin A Conjugates (Thermo Fisher Cat# A12379) for 1 h. The cytoskeleton was stained using l g/mL of Alexa Fluor^TM^ 488 Phalloidin (Thermo Fisher Cat# C21421) for 30 min. Nuclei were labeled using DAPI for 15 min. Images were acquired on a ZEISS fluorescence microscope using a 20× or 40× objective.

### Statistical Analysis

Quantitative data are presented as the mean ± the standard deviation (SD) from a minimum of three independent experiments. Comparisons between two groups were analyzed using Student’s *t*-test, unless otherwise indicated. Statistical analyses were performed with GraphPad Prism 6 (GraphPad Software Inc.). *p* < 0.05 was considered to be statistically significant.

## Results

### PERK Is Required for Myoblast Differentiation

**FIGURE 1:**
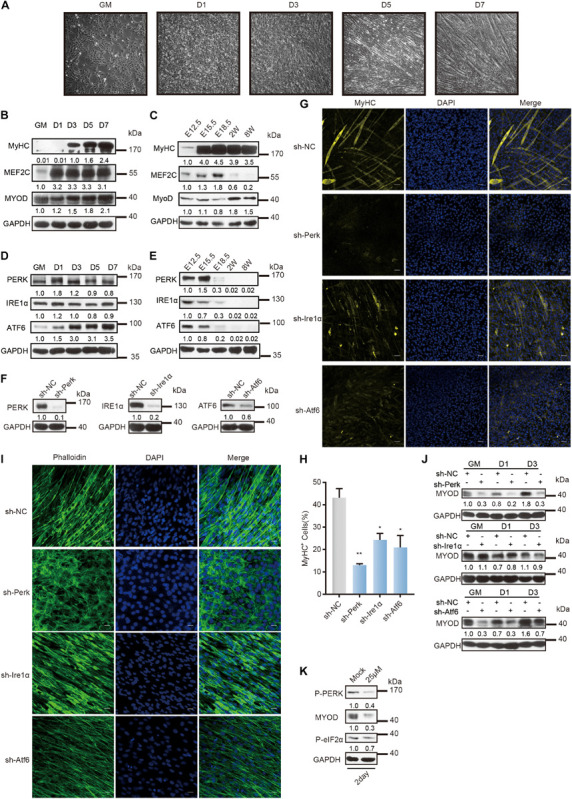
Distinct role of UPR sensors during myoblast differentiation. **(A)** Phase-contrast microscopy of differentiating C2C12 myoblasts in growth medium (GM) or 1, 3, 5, or 7 days in the differentiation medium. **(B)** Western blot analysis of skeletal muscle differentiation markers in whole-cell lysates from differentiating C2C12 myoblasts. **(C)** Western blot analysis of skeletal muscle differentiation markers in tissue lysates from developing mouse embryo muscle. Mouse hind limb muscles were isolated at five time points: E12.5, E15.5, E18.5, postnatal week 2, and postnatal week 8 (adult). Skeletal muscle development in mice consists of embryonic development (E8.5/9-E14.5), fetal development (E14.5-E19), perinatal development (P0–3/4 weeks), and adult development (3/4 week-aging). **(D)** Western blot analysis of the three UPR sensors in whole-cell lysates from differentiating C2C12 myoblasts. **(E)** Western blot analysis of the three UPR sensors in tissue lysates from developing mouse embryo muscle. **(F)** Western blot analysis of whole-cell lysates from PERK-, IRE1α-, or ATF6-knockdown cells and negative control cells to determine the knockdown efficiency. **(G)** Effects of PERK, IRE1α, or ATF6 knockdown on MyHC expression and myotube formation. PERK-, IRE1α-, or ATF6-knockdown cells and negative control cells were incubated in differentiation medium for 3 days and were then stained with an anti-myosin antibody and DAPI (nuclei). Scale bar: 50 μm. **(H)** Quantification of percentage of MyHC^+^ myotubes in wild-type and knockdown cells incubated in differentiation medium for 3 days. **(I)** Effects of PERK, IRE1α, or ATF6 knockdown on cell morphology changes. PERK-, IRE1α-, or ATF6-knockdown cells and negative control cells were incubated in differentiation medium for 3 days and were then fixed, permeabilized, and stained with phalloidin (F-actin) and DAPI (nuclei). Scale bar: 25 μm. **(J)** Western blots showing the effects of PERK, IRE1α, or ATF6 knockdown on the MyoD protein level. **(K)** Western blot analysis of MyoD in whole-cell lysates from C2C12 myoblasts under the optimal treatment time and PERK inhibitor (GSK2606414) concentration conditions. GAPDH was used as the internal control (A representative western blot is shown, *n* = 3.) The error bars indicate the mean ± standard deviation (SD) (*p < 0.05, **p < 0.01) from three independent experiments.

To establish a system for studying myoblast differentiation, we utilized C2C12 mouse skeletal myoblasts obtained from the skeletal muscles of myodystrophic mice, which are often used for studying myogenesis *in vitro* (Yaffe and Saxel, 1977). When C2C12 myoblasts proliferated to a high confluence in growth medium, we replaced the medium with the differentiation medium. In the early differentiation stage, C2C12 myoblasts proliferated to myocytes and gradually fused. During differentiation, myocytes fused to form multinucleated myofibers (Figure 1A). The protein expression levels of differentiation markers, such as MyoD, MEF2C, and Myosin heavy chain (MyHC) protein, showed signature profiles during C2C12 myoblast differentiation (Figure 1B), indicating a consistent trend of skeletal muscle differentiation during embryonic development (Figure 1C).

The three sensors of UPR are involved in the differentiation of C2C12 myoblasts, as detected by western blot and reverse transcription quantitative PCR (RT–qPCR) and shown in Figure 1D and Supplementary Figure 1A. Note that the expression of *Perk* was upregulated on the first day of differentiation and then decreased gradually. Changes in the expression of *Ire1*α were not evident, while the expression of *Atf6* was strongly upregulated on day three of differentiation. Western blot and RT-qPCR showed that the three UPR sensors were highly expressed during the embryonic stage and that their expression then declined (Figure 1E and Supplementary Figure 1B).

To investigate the important roles of the three UPR sensors in myoblast differentiation, we knocked down their expression separately by constructing three stable lentivirus-mediated C2C12 cell lines expressing a small hairpin RNA (shRNA) against *Perk* (shPerk), *Atf6* (shAtf6), or *Ire1*α (shIre1α) (Figure 1F and Supplementary Table 4). We compared these knockdown C2C12 myoblasts with wild-type myoblasts 3 days after induction and differentiation and found that each UPR protein is important for myoblast differentiation. As shown in Figures 1G,H, disruption of *Ire1*α or *Atf6* expression markedly reduced the expression of MyHC and myotube formation. More critically, disruption of *Perk* expression not only prevented the expression of MyHC and myotube formation but also affected cell morphology. We acquired images of cell morphology after phalloidin immunostaining for the cytoskeletal protein F-actin and found that PERK knockdown cells remained undifferentiated or even exhibited a rounded morphology, indicating that the PERK arm is a critical factor for myogenesis (Figure 1I).

Consistent with these results, western blot and RT-qPCR analyses revealed that knocking down each UPR sensor reduced the transcription and translation levels of *Myod* (Figure 1J and Supplementary Figure 1C). We found that knocking down IRE1α distinctly affected the MyoD protein level in C2C12 myoblasts only after differentiation was induced. Knocking down ATF6 immediately affected the MyoD protein level in C2C12 myoblasts before differentiation was induced, but the MyoD protein level in ATF6 knockdown cells recovered with differentiation. Importantly, knocking down PERK affected the MyoD protein level throughout the change in C2C12 myoblast fate. To further demonstrate the regulatory role of the PERK arm in *Myod* expression during myoblast differentiation, we treated C2C12 myoblasts with the PERK signaling pathway inhibitor GSK2606414, which inhibits the activation of the downstream pathway of PERK by inhibiting the autophosphorylation of PERK, at a concentration of 25 μM for 2 days (Supplementary Figure 1D). It was found that this high concentration of the PERK pathway inhibitor significantly inhibited the MyoD protein level (Figure 1K).

Taken together, these data indicated that the UPR has a crucial function in myogenesis, where PERK governs the initiation of myoblast differentiation and is required for the differentiation of myoblasts.

### Knocking Down PERK Induces Dedifferentiation of C2C12 Myoblasts by Altering the miRNA Network

**FIGURE 2:**
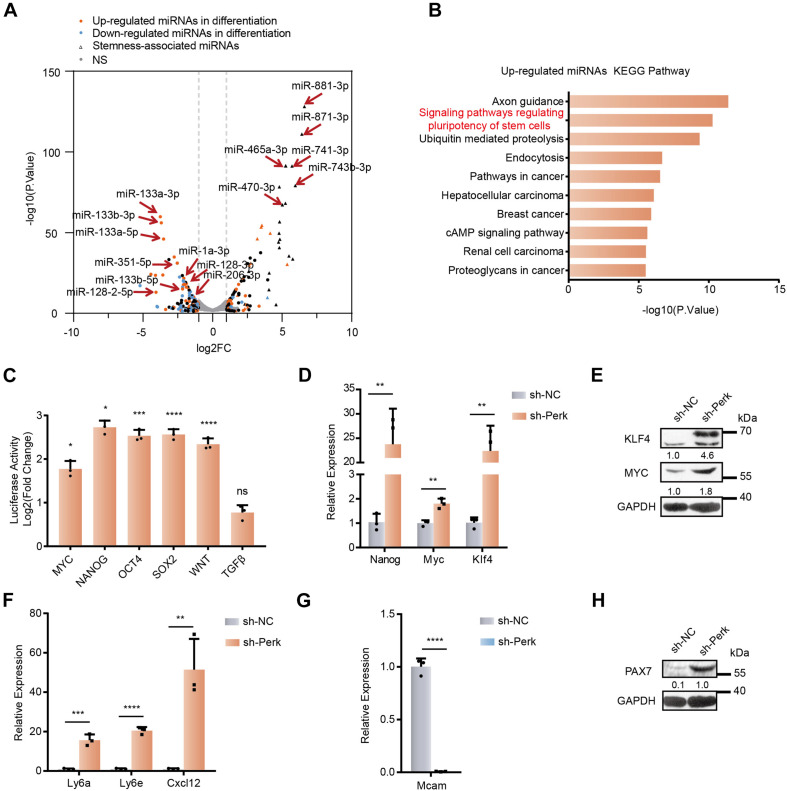
MicroRNA profiles and stemness changes in C2C12 myoblasts upon PERK knockdown. **(A)** A volcano plot of differentially expressed miRNAs in PERK knockdown cells. PERK knockdown cells and negative control cells were differentiated for 3 days. **(B)** KEGG analysis of upregulated miRNAs in PERK-knockdown cells. **(C)** Luciferase reporter assay showing the effects of PERK knockdown on the activity of stemness or differentiation pathways. The exponential calculation on the ratio was employed between each individual experimental value and the control value in their calculations of the luciferase data. **(D)** RT-qPCR was used to detect the effects of PERK knockdown on reprogramming factors. **(E)** Western blots showing the effects of PERK knockdown on the expression of reprogramming factors. **(F)** The relative mRNA expression levels of the stem cell markers *Ly6a*, *Ly6e*, and *Cxcl12* in PERK knockdown cells and negative control cells. **(G)** The relative mRNA expression levels of the myoblast marker *Mcam* in PERK knockdown cells and negative control cells. **(H)** Western blots showing the effects of PERK knockdown on the expression of the satellite cells marker PAX7. GAPDH was used as the internal control (A representative western blot is shown, *n* = 3.) The error bars indicate the mean ± standard deviation (SD) (*p < 0.05, **p < 0.01, ***p < 0.001, ****p < 0.0001) from three independent experiments.

As PERK knockdown cells remained undifferentiated but the change in cell morphology implied their altered status, we characterized the PERK knockdown cells by their molecular signature. miRNAs are a class of cell lineage determinants, which can also be regarded as molecular indicators of cell type or functional states (Xie et al., 2013). We first profiled miRNA expression by performing small RNA sequencing (RNA-seq) and analysis of PERK knockdown and negative control cells after 3 days of induction of differentiation. Knocking down PERK globally affected miRNA expression in myoblasts; more than 200 miRNAs were changed, among which 112 were significantly upregulated and 117 were significantly downregulated (Figure 2A and Supplementary Table 1). The most significantly changed miRNAs were verified by RT-qPCR, and the results were consistent with the sequencing results (Supplementary Figure 2A). Interestingly, compared with the differentially expressed miRNAs in C2C12 cells differentiated for 3 days, some upregulated miRNAs involved in skeletal muscle differentiation, including many myogenesis-associated miRNAs, such as miR-133a, miR-133b, miR-206, miR-1a, and miR-128, were downregulated in PERK knockdown cells (Figure 2A and Supplementary Table 2). In contrast, many stemness-related miRNAs were upregulated in PERK knockdown cells (Figure 2A and Supplementary Table 2). Surprisingly, a class of X-linked miRNAs exist in the fragile X region of the X chromosome of placental mammals and perform an important regulatory function in the transition from proliferation to maturity during spermatogenesis; for example, miR-881, miR-871, miR-741, miR-465, miR-470, and miR-743b were significantly upregulated in PERK knockdown cells (Figure 2A and Supplementary Table 2) (Ramaiah et al., 2019). Among these miRNAs, miR-470 has been identified as a miRNA that is highly expressed in mouse embryonic stem cells (Tay et al., 2008). Comparison of the differential miRNA expression between PERK knockdown cells and differentiated C2C12 cells indicated that knocking down PERK impacts the state of myoblast differentiation by changing miRNA expression.

KEGG pathway analysis of the targets of the changed miRNAs further showed that the functions of the upregulated miRNAs (e.g., signaling pathway regulating pluripotency of stem cells) were related to the pluripotent state (Figure 2B). We further analyzed the activity of the signaling pathways associated with stemness in PERK knockdown cells by using the Qiagen’s Cignal pathway reporter vector and dual fluorescence reporter system. Knocking down PERK markedly upregulated the activity of stemness-related signaling pathways, such as the Nanog, Wnt, Myc, Sox2, and Oct4 signaling pathways; in contrast, the TGFβ pathway, which promotes cell transdifferentiation, had no significant change (Figure 2C and Supplementary Figure 2B) (Cartwright et al., 2005). Meanwhile, knocking down PERK enhanced the expression of the two reprogramming factors *Myc* and *Klf4* (Figures 2D,E). The transcription level of the pluripotent stem cell marker *Nanog* was also significantly upregulated by more than 20-fold (Figure 2D). We further confirmed that PERK knockdown significantly promoted the stemness of cells by detecting the transcriptional level of lymphocyte antigen 6a/e (*Ly6a/e*), which is highly expressed in various progenitor cells during the differentiation and development of mesoderm cells (Figure 2F) (van de Rijn et al., 1989; Van Vlasselaer et al., 1994; Welm et al., 2002; van Bragt et al., 2005). Additionally, knocking down PERK dramatically enhanced the transcriptional level of C–X–C motif chemokine ligand 12 (*Cxcl12*), which maintains the stem cell characteristics of bone marrow mesenchymal stem cells (Figure 2F) (Peled et al., 1999; Janssens et al., 2018; Matsushita et al., 2020). Notably, PERK suppression also significantly impaired the characteristics of C2C12 myoblast, as we determined by assessing the transcriptional level of melanoma cell adhesion molecule (*Mcam*), which is highly expressed in proliferating myoblasts and significantly downregulated during fusion (Figure 2G) (Lapan and Gussoni, 2012; Alexander et al., 2016). PAX7, a marker of satellite cells, was obviously upregulated in PERK knockdown cells, which further demonstrated that dedifferentiation of myoblasts into satellite cells is truly occurring (Figure 2H and Supplementary Figure 2C). In order to further verify the effects of PERK knockdown on the characteristics of cells, we used cloning and CCK-8 to detect the proliferation rate of PERK knockdown cells and found that the proliferation rate of PERK knockdown cells was reduced (Supplementary Figures 2D,E).

Together, these findings indicated that knocking down PERK induced dedifferentiation of myoblasts into stem-like cells by effectively altering key miRNAs and their networks in cell fate determination. These results demonstrated that PERK plays an important role in the formation, maintenance, and differentiation of myoblasts.

### The PERK-ATF4 Signaling Pathway Transactivates Differentiation-Associated miRNAs in the Early Stage of Myoblast Differentiation

**FIGURE 3:**
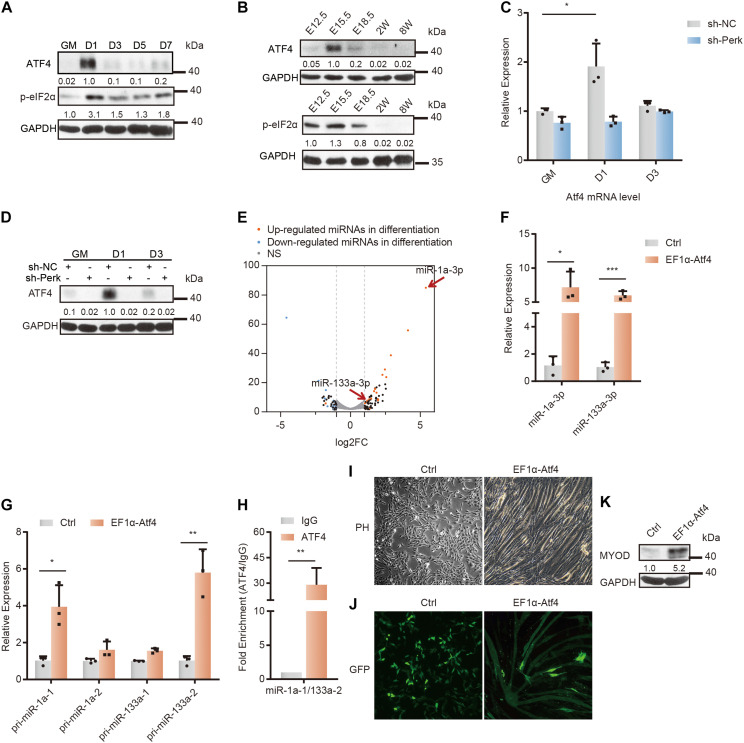
Changes in the microRNA profile and differentiation of C2C12 myoblasts upon ATF4 overexpression. **(A)** Western blot analysis of p-eif2α and ATF4 in whole-cell lysates from differentiating C2C12 myoblasts. **(B)** Western blot analysis of p-eif2α and ATF4 in tissue lysates from developing mouse embryo muscle. **(C)** RT-qPCR was used to detect the effects of PERK knockdown on the transcription level of *Atf4*. **(D)** Western blots showing the effects of PERK knockdown on the ATF4 protein level. **(E)** A volcano plot of differentially expressed miRNAs in ATF4-overexpressing cells. ATF4-overexpressing cells and negative control cells were incubated in growth medium and allowed to proliferate to 80% confluence. **(F)** The relative expression levels of miR-1a-3p and miR-133a-3p in ATF4-overexpressing cells and negative control cells. **(G)** The relative expression levels of primary transcripts of miR-1a and miR-133a in ATF4-overexpressing cells and negative control cells. **(H)** ChIP-qPCR for validation of the ATF4 binding sites in the common promoter region of miR-1a-1 and miR-133a-2. **(I)** Phase-contrast microscopy of ATF4-overexpressing cells or negative control cells in growth medium showing the effects of ATF4 overexpression on myoblast differentiation and myotube formation. **(J)** Fluorescence microscopy of ATF4-overexpressing cells or negative control cells transiently transfected with the same amount of monomeric green fluorescent protein (mGFP) in growth medium showing the effects of ATF4 overexpression on myoblast differentiation and myotube formation. Normal myoblasts do not have enough space to continue to grow after the confluence of the culture reaches 100%, so they must be passaged. **(K)** Western blots showing the effects of ATF4 overexpression on the expression of Myod. GAPDH was used as the internal control (A representative western blot is shown, *n* = 3). The error bars indicate the mean ± standard deviation (SD) (*p < 0.05, **p < 0.01, ***p < 0.001) from three independent experiments.

It is well known that activation of PERK can reduce general translational initiation through eIF2α phosphorylation, which can specifically activate the translation of *Atf4*. ATF4 is the major downstream transcriptional activator of the PERK-eIF2α pathway and may direct the induction of specialized transcriptomes including both coding and non-coding RNAs in response to various physiological stresses (Hinnebusch and Natarajan, 2002; Ron and Walter, 2007; Wang et al., 2009). To investigate whether PERK regulates miRNA expression by activating ATF4 in myoblasts, we first examined the expression of *Atf4* during myoblast differentiation and compared it with the expression pattern of its upstream PERK-eIF2α signaling mediator phosphorylated eIF2α. Immediately after the differentiation of C2C12 myoblasts was induced, ATF4 was markedly upregulated and then rapidly downregulated, consistent with the activation of eIF2α phosphorylation both *in vitro* and *in vivo* (Figures 3A,B and Supplementary Figures 3A,B), indicating that ATF4 plays an important role in the early stage of myoblast differentiation. We further confirmed that PERK knockdown inhibited the transcriptional activity of ATF4 by ATF4 signaling pathway reporter vector and dual fluorescence reporter assays (Supplementary Figure 3C). The western blot and RT-qPCR results further verified that PERK knockdown suppressed the ATF4 protein level but only slightly affected its transcription (Figures 3C,D).

To study the relationship between the ATF4 activation and miRNA expression, we constructed an ATF4 overexpression vector and introduced it into C2C12 cells (Supplementary Figures 3D,E, and Supplementary Table 4). Small RNA-seq and analysis showed that ATF4 overexpression partially regulated the differentiation-related miRNA expression profile in myoblasts under undifferentiation conditions (Figure 3E). Evidently, miR-133a and miR-1a, specific miRNAs for skeletal muscle differentiation, were upregulated in ATF4-overexpressing cells (Figures 3E,F and Supplementary Table 3). This typical change in the miRNA profile strongly suggests that ATF4, the PERK-activating transcription factor, can promote myoblast differentiation by regulating miRNA networks.

Furthermore, the primary transcripts of miR-133a-2 and miR-1a-1 were verified to be upregulated by ATF4 overexpression (Figure 3G). Subsequently, as miR-133a-2 and miR-1a-1 are derived from the same polycistronic miRNA and co-transcribed, we performed ChIP with an anti-ATF4 antibody in ATF4-overexpressing cells to further determine that ATF4 can regulate the transcription of miR-133a-2 and miR-1a-1 (Chen et al., 2006). By searching for potential ATF4-binding sites in the promoter region of these specific miRNA genes and designing qPCR primers for ChIP-qPCR, we found substantial binding of ATF4 in the promoter region of miR-1a-1 and miR-133a-2, indicating that ATF4 directly bound to the promoter region of these specific miRNA genes to promote their expression (Figure 3H).

As a result of differentiation initiation, ATF4 overexpression obviously impaired the proliferation of C2C12 myoblasts (Supplementary Figure 3F). Moreover, ATF4-overexpressing cells fused with each other in the growth medium not supplemented with any pro-differentiation factors (Figure 3I). In the growth medium, the fluorescent protein GFP was clearly expressed in myotube-like fused ATF4-overexpressing cells, illustrating that ATF4 can facilitate cell fusion and myoblast differentiation (Figure 3J). The RT-qPCR results further verified that ATF4 overexpression significantly increased the transcriptional level of myoblast fusion factors myomaker (*Mymk*) and myomixer (*Mymx*) (Supplementary Figure 3G) (Millay et al., 2013; Leikina et al., 2018). Importantly, overexpression of only ATF4 increased the MyoD protein level, which demonstrated that ATF4 can activate the differentiation program in myoblasts (Figure 3K).

Taken together, these results indicated that ATF4 facilitated the expression of a set of differentiation-associated miRNAs. This finding suggests that the PERK-ATF4 pathway is the major signaling pathway for miRNA regulation during myoblast differentiation and that ATF4, as a PERK-activating transcription factor, plays a key role in cell differentiation and fate determination.

### A Positive Feedback Loop Is Formed Between PERK-Regulated miR-128 and UPR by Regulating Ppp1cc in Myoblast Differentiation

**FIGURE 4:**
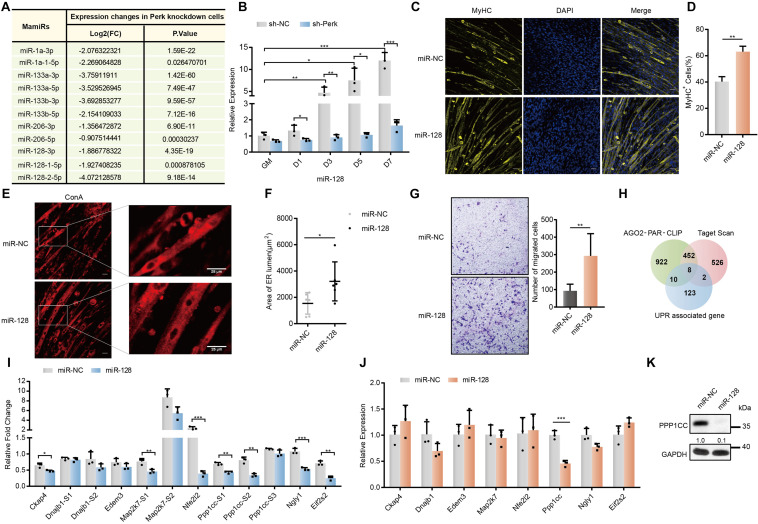
Screening of potential targets for miR-128 regulated by PERK. **(A)** Changes in the expression of mamiRs in PERK knockdown cells. **(B)** The relative expression levels of miR-128 in PERK knockdown cells and negative control cells. **(C)** Effects of miR-128 overexpression on MyHC expression and myotube formation. miR-128-overexpressing cells and negative control cells were incubated in differentiation medium for 3 days and were then stained with an anti-myosin antibody and DAPI (nuclei). Scale bar: 50 μm. **(D)** Quantification of percentage of MyHC^+^ myotubes in wild-type and miR-128-overexpressing cells incubated in differentiation medium for 3 days. **(E)** Effects of miR-128 overexpression on endoplasmic reticulum expansion. miR-128-overexpressing cells and negative control cells were incubated in differentiation medium for 3 days and were then fixed, permeabilized, and stained with Concanavalin A (endoplasmic reticulum). Scale bar: 25 μm. **(F)** The area of ER lumen was quantified by measuring the area of the ER lumen stained with Concanavalin A in the photographs. Values for the area of ER lumen from chosen two multinucleated myofibers of each photograph. **(G)** Cell migration assay showing the effects of miR-128 overexpression on myoblast migration. **(H)** Venn diagram showing the proportion of UPR-associated genes targeted by miR-128 as indicated by AGO2-PAR-CLIP or potentially targeted by miR-128 as indicated by TargetScan database analysis. **(I)** Luciferase reporter assay showing the effects of miR-128 on the wild-type (WT) or mutant (MT) reporter constructs. All luciferase activity levels were normalized to those of firefly luciferase driven by a single promoter in the reporter vector. **(J)** qRT-PCR assays showing the effects of miR-128 overexpression on potential targets. **(K)** Western blots showing the effects of miR-128 overexpression on the potential target *Ppp1cc.* GAPDH was used as the internal control (A representative western blot is shown, *n* = 3). The error bars indicate the mean ± standard deviation (SD) (*p < 0.05, **p < 0.01, ***p < 0.001) from three independent experiments.

To further identify the miRNAs affected by the PERK signaling pathway during myoblast differentiation, we first identified significantly downregulated miRNAs in PERK knockdown cells by using small RNA-seq data. Unsurprisingly, myogenesis-associated miRNAs (mamiRs) that play important roles in signaling pathways were identified (Figure 4A) (Xie et al., 2018). As miR-128 was one of the most markedly downregulated mamiRs in PERK knockdown myoblasts (Figures 4A,B), we next investigated whether miR-128 is deeply implicated in myoblast differentiation. Immunofluorescence staining for MyHC showed that miR-128 promoted myoblast differentiation and myotube formation (Figures 4C,D). In addition, the larger size of the ER in the miR-128-overexpressing cells was corroborated by labeling the differentiated C2C12 myoblasts with the endoplasmic reticulum-specific probe concanavalin A (Figures 4E,F), implying a potential functional mechanism of miR-128 in the UPR. Furthermore, miR-128 promoted cell migration, a necessary step in the early differentiation of myoblasts, which requires greatly increased cell surface protein synthesis in the endoplasmic reticulum (Figure 4G). The transcriptional level of the myoblast fusion factors *Mymk* and *Mymx* was also significantly upregulated in miR-128-overexpressing cells (Supplementary Figures 4A,B). In addition, miR-128 overexpression immediately increased the expression of *Myod* after the differentiation of C2C12 myoblasts was induced (Supplementary Figures 4C,D). These observations indicated that miR-128 is involved in the early phase of myoblast differentiation by regulating UPR-associated physiological functions.

To further investigate the potential targets of miR-128 related to the UPR, we combined UPR-associated genes with predicted miR-128 targets from our previous Ago2-PAR-CLIP data and TargetScan database analysis and found that the mRNAs of multiple factors related to the UPR may be regulated by miR-128 (Figure 4H) (Xie et al., 2018). We introduced these potential targets and important regulators of the UPR pathway into String online software to explore their functional relationships and found that *Ppp1cc*, *Nfe2l2*, and *Map2k7* are closely related to the UPR pathway (Supplementary Figure 4E). To verify the interactions between miR-128 and its potential targets, we constructed Renilla luciferase reporter vectors containing either a wild-type or mutant binding site for the potential target and transfected them into C2C12 cells. Among the potential targets we selected, miR-128 had a relatively high ability for direct binding to the target sites in *Ckap4*, *Ppp1cc*, *Map2k7*, *Nfe2l2*, *Eif2s2*, and *Ngly1* (Figure 4I). miR-128 significantly decreased the mRNA level of *Ppp1cc* in this group of potential targets (Figure 4J). The PPP1CC protein level was also markedly inhibited by miR-128, further verifying that miR-128 can potently block endogenous translation of *Ppp1cc* (Figure 4K).

**FIGURE 5:**
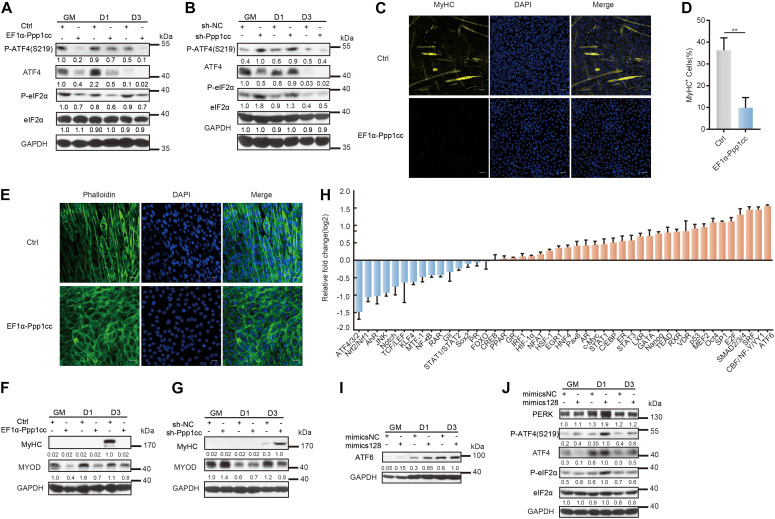
Ppp1cc is the target of miR-128 in a positive feedback loop regulating the UPR during C2C12 myoblast differentiation. **(A)** Western blots showing the effects of PPP1CC overexpression on the p-eIF2α-ATF4 signaling pathway during C2C12 myoblast differentiation. **(B)** Western blots showing the effects of PPP1CC knockdown on p-eIF2α-ATF4 signaling pathway during C2C12 myoblast differentiation. **(C)** Effects of PPP1CC overexpression on MyHC expression and myotube formation. PPP1CC-overexpressing cells and negative control cells were incubated in differentiation medium for 3 days and were then stained with an anti-myosin antibody and DAPI (nuclei). Scale bar: 50 μm. **(D)** Quantification of percentage of MyHC^+^ myotubes in wild-type and PPP1CC-overexpressing cells incubated in differentiation medium for 3 days. **(E)** Effects of PPP1CC overexpression on the morphology of myoblasts. PPP1CC-overexpressing cells and negative control cells were incubated in differentiation medium for 3 days and were then fixed, permeabilized, and stained with Phalloidin (F-actin) and DAPI (nuclei). Scale bar: 25 μm. **(F)** Western blots showing the effects of PPP1CC overexpression on the protein level of MyHC and MyoD during C2C12 myoblast differentiation. **(G)** Western blots showing the effects of PPP1CC knockdown on the protein level of MyHC and MyoD during C2C12 myoblast differentiation. **(H)** Luciferase reporter assay showing the effects of miR-128 overexpression on the activity of signaling pathways. The exponential calculation on the ratio was employed between each individual experimental value and the control value in their calculations of the luciferase data. **(I)** Western blots showing the effects of miR-128 overexpression on the ATF6 protein level during C2C12 myoblast differentiation. **(J)** Western blots showing the effects of miR-128 overexpression on the p-eIF2α-ATF4 signaling pathway during C2C12 myoblast differentiation. GAPDH was used as the internal control (A representative western blot is shown, *n* = 3). The error bars indicate the mean ± standard deviation (SD) (**p < 0.01) from three independent experiments.

Of note, the primary regulatory mechanism for the ATF4 protein level depends on the p-eIF2α-mediated translational mechanism, which is destabilized by a catalytic subunit (PP1) and a regulatory subunit, either GADD34 induced by ATF4 or the constitutively expressed paralogue CReP (Novoa et al., 2001). To study the relationship between PPP1CC and myoblast differentiation, we further generated stable PPP1CC overexpression and PPP1CC knockdown C2C12 cells (Supplementary Figures 5A,B and Supplementary Table 4). PPP1CC overexpression inhibited the phosphorylation of eIF2α and reduced the expression of total ATF4, but PPP1CC knockdown promoted the phosphorylation of eIF2α without the increase in the total ATF4 protein level resulting from the increased phosphorylation level of ATF4 (Figures 5A,B), indicating that PPP1CC can regulate the activity of the p-eIF2α-dependent signaling pathway. Unexpectedly, PPP1CC overexpression strikingly prevented the expression of MyHC and myotube formation (Figures 5C,D). Meanwhile, we photographed their cell morphology and found that PPP1CC-overexpressing cells remained undifferentiated, with a morphology similar to the rounded morphology of PERK knockdown cells (Figure 5E). Western blot analysis further verified that PPP1CC overexpression led to a decrease in protein levels of MyHC and MyoD (Figure 5F). However, knocking down PPP1CC could promote levels of MyoD in C2C12 myoblasts that were incubated in the growth medium (Figure 5G). Considering this result, we determined that inhibition of Ppp1cc by miR-128 is crucial for myoblast differentiation.

To further verify the mechanism by which miR-128 regulates the UPR signaling pathway, we used the Cignal 45 signaling pathway reporting system to analyze the function of miR-128 in myoblasts. Importantly, the signaling pathway most significantly upregulated by miR-128 was the ATF6 pathway, while the pathway most obviously downregulated by miR-128 was the ATF4 pathway (Figure 5H). We found miR-128 promoted the ATF6 protein level in C2C12 myoblasts on the first day of induced differentiation (Figure 5I). Intriguingly, miR-128 immediately suppressed the total ATF4 protein level in the growth medium, but this effect was impaired when myoblasts were induced to differentiate (Figure 5J). Once myoblasts were induced to differentiate, miR-128 promoted the phosphorylation of eIF2α and further enhanced the translation of *Atf4*, but the phosphorylation of ATF4 on S219 was simultaneously promoted (Figure 5J). It has been reported that the f-box protein bTrCP, the receptor component of the E3 ubiquitin ligase SCF, can co-localize with ATF4 in the nucleus and bTrCP can control its stability by identifying the phosphorylated S219 site on ATF4 to promote the degradation of ATF4 (Lassot et al., 2001). The phosphatase PP1 is implicated in promoting the translation of *Atf4* and dephosphorylating ATF4 on S219, which is in turn implicated in regulating ATF4 to control the total ATF4 protein level in a time- and dose-dependent manner (Frank et al., 2010). The above experimental results indicate that miR-128 can regulate the activity of the PERK-eIF2α pathway by a positive feedback regulation of eIF2α phosphorylation and further promote the degradation of its downstream effector ATF4 by its phosphorylation on S219 to maintain the ATF4 protein level within a certain range and allow it to perform its functions within this range (Supplementary Figure 5C).

Together, our results suggest that PERK-regulated miR-128 is important for myoblast differentiation and that a positive feedback loop is formed between miR-128 and the UPR via the phosphatase subunit PPP1CC during myogenesis.

### ARPP21 Antagonizes miR-128 Activity by Co-regulating the Phosphatase Subunit PPP1CC During Myoblast Differentiation

**FIGURE 6:**
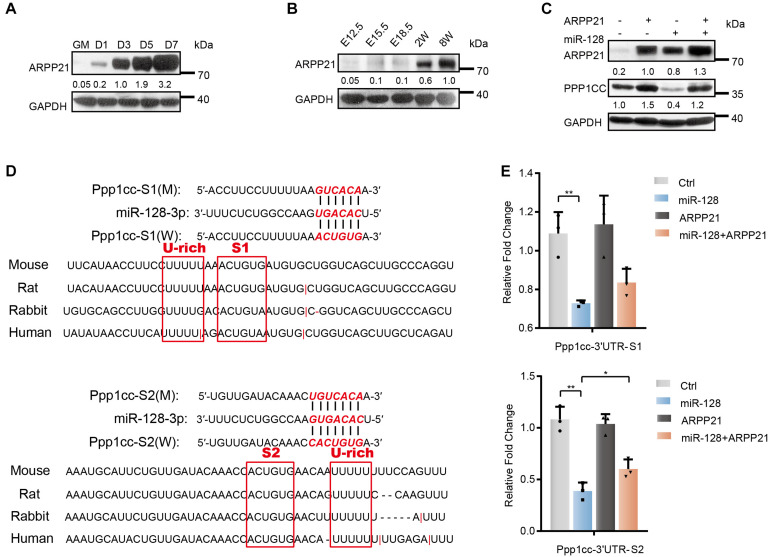
Antagonistic effect of ARPP21 and miR-128 on Ppp1cc mRNA. **(A)** Western blot analysis of ARPP21 in whole-cell lysates from differentiating C2C12 myoblasts. **(B)** Western blot analysis of ARPP21 in tissue lysates from developing mouse embryo muscle for ARPP21. **(C)** Western blots showing PPP1CC protein levels. ARPP21 leads to increased protein expression of PPP1CC. miR-128 leads to reduced expression of PPP1CC. ARPP21 rescued the inhibitory effects of miR-128 on *Ppp1cc*. **(D)** Sequence analysis of the *Ppp1cc* 3′UTR. **(E)** Luciferase reporter assay results showing the effects of miR-128 and ARPP21 on the 3′UTR reporter constructs. GAPDH was used as the internal control (A representative western blot is shown, *n* = 3). The error bars indicate the mean ± standard deviation (SD) (*p < 0.05, **p < 0.01) from three independent experiments.

ARPP21, encoding by the host gene of miR-128-2, has been implicated in different aspects of posttranscriptional regulation by binding mRNAs and interacting with the translation initiation complex eIF4F to prevent its association with uridine-rich elements in the 3′UTRs of miR-128 target genes during neural development (Rehfeld et al., 2018). To study the endogenous function of ARPP21 during myoblast differentiation, we first evaluated the expression of *Arpp21*. As shown, the ARPP21 protein level was markedly upregulated during the differentiation of C2C12 myoblasts (Figure 6A). We also examined levels of ARPP21 in skeletal muscle tissue samples from mouse embryos to determine whether the expression pattern described above is related to the *in vivo* function of ARPP21 in the context of organism development (Figure 6B). To further demonstrate whether the miR-128-mediated repression of *Ppp1cc* mRNA can be alleviated by ARPP21, we first detected the PPP1CC protein level in stable ARPP21-overexpressing cells (Figure 6C). We found that ARPP21 overexpression increased the PPP1CC protein level. Importantly, this effect was reversed by exogenous expression of miR-128 mimics, which led to a marked reduction in the PPP1CC protein level. As shown in Figure 6D, the uridine-rich element was adjacent to the miR-128 seed sequence in the 3′UTR of *Ppp1cc*. To further investigate the role of the *Ppp1cc* 3′UTR in ARPP21-mediated regulation, we constructed a Renilla luciferase (RL) reporter vector containing segments of the *Ppp1cc* 3′UTR. The reporter activity was normalized to the activity of co-expressed firefly luciferase (FL). We found that treatment of ARPP21-overexpressing cells with miR-128 mimics, preventing the repression of RL-Ppp1cc-S2 mRNA by miR-128, abolished the effects of ARPP21 on the activity of this reporter (Figure 6E).

Together, the above experiments indicate that the progressive ARPP21 during myoblast differentiation can reduce the inhibitory effects of miR-128 on *Ppp1cc*, negatively regulate miR-128, and avoid the constitutive UPR activity mediated by miR-128. This mechanism is consistent with the inactivation of stress responses, including the UPR, when myoblasts enter homeostasis at a later stage of differentiation.

## Discussion

**FIGURE 7:**
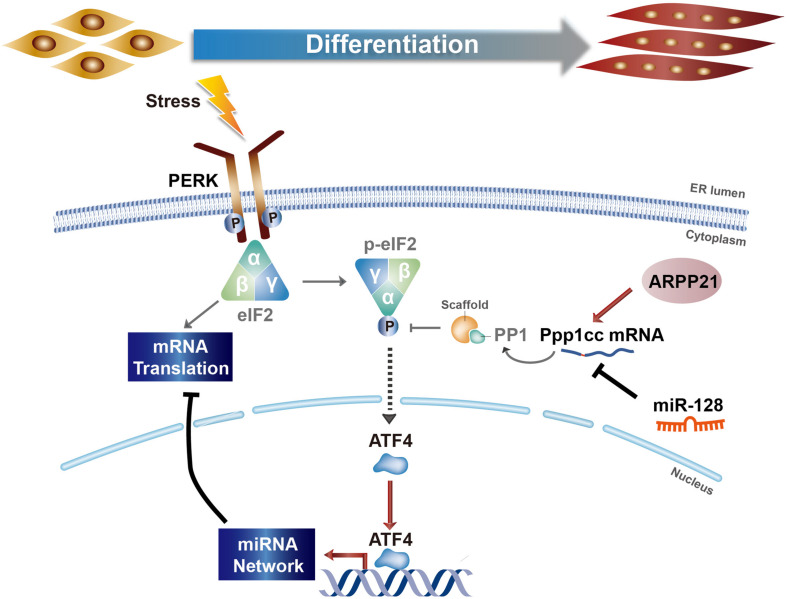
Model of the regulatory relationship between PERK and miRNA networks during myoblast differentiation.

Our study reveals the mechanism of PERK signaling in regulating miRNA networks during myoblast differentiation. A schematic of the molecular regulatory mechanism is shown in Figure 7. In the early stage of myoblast differentiation, stress responses, including the UPR, are activated in myoblasts. Once the PERK signaling pathway, one arm of the UPR, is activated, the downstream effector-phosphorylated eIF2α immediately promotes the translation of mRNAs with uORF structures, producing a large number of specific proteins, including ATF4. ATF4 can enter the nucleus, further activating the downstream transcription of specific genes, including many miRNA genes. Our study verified that ATF4, as a downstream effector of PERK signaling, can directly promote the expression of myomiRs, such as miR-1a and miR-133a, in myoblasts. Subsequently, miRNAs activated by the PERK signaling pathway can regulate the posttranscriptional level of a large number of mRNAs in the cytoplasm and inhibit their translation, resulting in dynamic changes in the proteome during myoblast differentiation. In addition, PP1 phosphatase catalytic subunit PPP1CC, the main regulatory protein of p-eIF2α dephosphorylation, was inhibited by PERK-regulated miR-128 during myoblast differentiation, demonstrating that a novel mechanism involving a positive feedback loop between miRNAs and the UPR is active in promoting myoblast differentiation in the early stage. Intriguingly, this mechanism not only positively regulates the functional activity of phosphorylated eIF2α but also promotes the phosphorylation and degradation of ATF4 so that the ATF4 protein level is maintained within a certain range. Along with the process of differentiation, the RNA-binding protein ARPP21, the product of the miR-128-2 host gene *Arpp21*, can antagonize the function of miR-128, thus promoting the expression of *Ppp1cc*, weakening the activity of the PERK-eIF2α signaling pathway and maintaining the homeostasis of myoblast differentiation.

PERK can affect miRNA networks associated with stemness and differentiation to determine the fate of myoblast differentiation, and it is interesting to determine whether this is a common mechanism in the process of cell differentiation. We therefore selected P19 mouse embryonic carcinoma cells for further research. We profiled miRNA expression in PERK knockdown P19 cells and negative control cells and found that knocking down PERK significantly affected the expression of a set of miRNAs, including 103 significantly upregulated miRNAs and 113 significantly downregulated miRNAs (Supplementary Figures 6A,B and Supplementary Table 9). The miRNA expression profile of PERK knockdown P19 cells was compared with those of undifferentiated P19 cells and P19 cells induced to differentiate into neurons for 4 days (Supplementary Table 10) (Huang et al., 2009). As expected, many miRNAs upregulated in P19 cell differentiation were downregulated in PERK knockdown P19 cells. KEGG pathway analysis of the targets of these differentially expressed miRNAs showed that the upregulated miRNAs were related to the pluripotent state (e.g., signaling pathway regulating pluripotency of stem cells) (Supplementary Figure 6D). We further determined that PERK suppression significantly upregulated the activity of stemness-related signaling pathways, such as the Nanog signaling pathway (Supplementary Figure 6F). Western blot analysis also showed that PERK knockdown significantly enhanced the NANOG protein level (Supplementary Figure 6E). These observations and analysis indicated that PERK regulates cell differentiation and fate determination through miRNA networks in different kinds of cells.

The UPR is a highly conserved signaling pathway that maintains endoplasmic reticulum homeostasis during the developmental process. Many studies have shown that the downstream effectors regulated by the three UPR sensors (PERK, IRE1, and ATF6) can affect the transcription and translation of a series of genes and participate in the regulation of cell growth, differentiation, and stemness maintenance. Our study reveals that PERK signaling acts as the main regulatory factor in the fate determination of myoblasts through regulating miRNA networks, further expanding our understanding of PERK signaling pathways in cell differentiation and biological development.

## Data Availability Statement

Small RNA sequencing data that support the findings of this study have been deposited in the Gene Expression Omnibus (GEO) under accession codes GSE167878.

## Ethics Statement

The animal study was reviewed and approved by Institutional Animal Care and Use Committee of Sun Yat-sen University.

## Author Contributions

L-HQ supervised the project. L-HQ, Y-YT, and YZ designed the experiments. Y-YT, YZ, S-JX, and P-PC performed the experiments. Y-YT, YZ, BL, L-LZ, S-QM, and Q-JH analyzed the data. L-HQ, Y-YT, YZ, and Y-WO wrote the manuscript. All authors contributed to the article and approved the submitted version.

## Conflict of Interest

The authors declare that the research was conducted in the absence of any commercial or financial relationships that could be construed as a potential conflict of interest.
