# Supplementary material for: PERK Signaling Controls Myoblast Differentiation by Regulating MicroRNA Networks
Source: Front Cell Dev Biol. 2021 May 28;9:670435. doi: 10.3389/fcell.2021.670435 (PMC8193987; doi:10.3389/fcell.2021.670435)
Supplement: Supplementary file 11 [file Data_Sheet_1.pdf]

TITLES AND LEGENDS TO SUPPLEMENTARY FIGURES

Figure S1. Effects of UPR sensors knockdown during myoblast differentiation

(A) The relative mRNA expression level of the three UPR sensors in differentiating C2C12 myoblasts.

(B) The relative mRNA expression levels of the three UPR sensors in developing mouse embryo muscle.

(C) The relative mRNA expression levels of *Myod* in PERK-, IRE1α- or ATF6-knockdown cells and negative control cells.

(D) Concentration and time determination of PERK inhibitor (GSK2606414) on C2C12 myoblasts. Western blot analysis of p-PERK and p-eIF2α in whole-cell lysates from C2C12 myoblasts under six different treatment concentration: 0 μM, 2.5 μM, 5 μM, 10 μM, 15 μM, 25 μM. Western blot analysis of p-PERK and p-eIF2α in whole-cell lysates from C2C12 myoblasts under four different treatment time: 0 day, 2 days, 4 days, 6 days.

GAPDH was used as the internal control. (A representative western blot is shown, n=3.) The error bars indicate the mean ± standard deviation (SD) (*$p < 0.05$) from three independent experiments.

Figure S2. Validation of differentially expressed miRNAs in C2C12 myoblasts upon PERK knockdown.

(A) RT-qPCR was used to verify the upregulated and downregulated miRNAs randomly selected in PERK-knockdown cells and negative control.

(B) Luciferase reporter assay showing the effects of PERK knockdown on relative luciferase activity of each stemness or differentiation pathway.

(C) Western blot analysis of the satellite cells marker PAX7 in tissue lysates from mouse foetal and perinatal muscle.

(D) Colony formation assay showing the effects of PERK knockdown on the proliferation of C2C12 myoblasts.

(E) CCK-8 assay showing the effects of PERK knockdown on the proliferation of

C2C12 myoblasts.

GAPDH was used as the internal control. (A representative western blot is shown, n=3.) The error bars indicate the mean ± standard deviation (SD) (*p < 0.05) from three independent experiments.

Figure S3. Effects of ATF4 overexpression on myoblast differentiation.

(A) The relative mRNA expression levels of *Atf4* in differentiating C2C12 myoblasts.

(B) The relative mRNA expression levels of *Atf4* in developing mouse embryo muscle.

(C) Luciferase reporter assay showing the effects of PERK knockdown on the activity of ATF4 signalling pathway.

(D) RT-qPCR was used to detect the effects of ATF4 overexpression on the transcription level of *Atf4*.

(E) Western blot analysis of whole-cell lysates from ATF4-overexpressing cells and negative control cells to determine the overexpression effciency.

(F) Colony formation assay showing the effects of ATF4 overexpression on the proliferation of C2C12 cells.

(E) RT-qPCR was used to detect the effects of ATF4 overexpression on the transcriptional level of fusion factors.

GAPDH was used as the internal control. (A representative western blot is shown, n=3.) The error bars indicate the mean ± standard deviation (SD) (*p < 0.05) from three independent experiments.

Figure S4. Effects of miR-128 overexpression on myoblast differentiation

(A) and (B) RT-qPCR was used to detect the effects of miR-128 overexpression on the transcriptional level of fusion factors.

(C) Western blots showing the effects of miR-128 overexpression on MyoD.

(D) RT-qPCR was used to detect the effects of miR-128 overexpression on the transcription level of Myod.

(E) Schematic diagram of functional relationship of potential targets analysized by

String.

GAPDH was used as the internal control. (A representative western blot is shown, n=3.) The error bars indicate the mean ± standard deviation (SD) (*p < 0.05) from three independent experiments.

Figure S5. Regulatory mechanism of miR-128 in p-eIF2α-ATF4 signalling pathway.

(A) Western blot analysis of whole-cell lysates from PPP1CC knockdown cells and negative control cells to determine the knockdown effciency.

(B) Western blot analysis of whole-cell lysates from PPP1CC-overexpressing cells and negative control cells to determine the overexpression effciency.

(C) Schematic diagram of feedback regulation for PERK pathway by miR-128.

GAPDH was used as the internal control. (A representative western blot is shown, n=3.)

Figure S6. MicroRNA profiles and stemness changes in P19 cells upon PERK knockdown.

(A) Western blot analysis of whole-cell lysates from PERK-knockdown P19 cells and negative control cells to determine the knockdown effciency.

(B) A volcano plot of differentially expressed miRNAs in PERK knockdown P19 cells.

(C) and (D) KEGG analysis of differentially expressed miRNAs in PERK knockdown P19 cells.

(E) Western blots showing the effects of PERK knockdown on the expression of reprogramming factors.

(F) RT-qPCR was used to detect the effects of PERK knockdown on reprogramming factors.

GAPDH was used as the internal control. (A representative western blot is shown, n=3.) The error bars indicate the mean ± standard deviation (SD) (*p < 0.05) from three independent experiments.

Figure S1

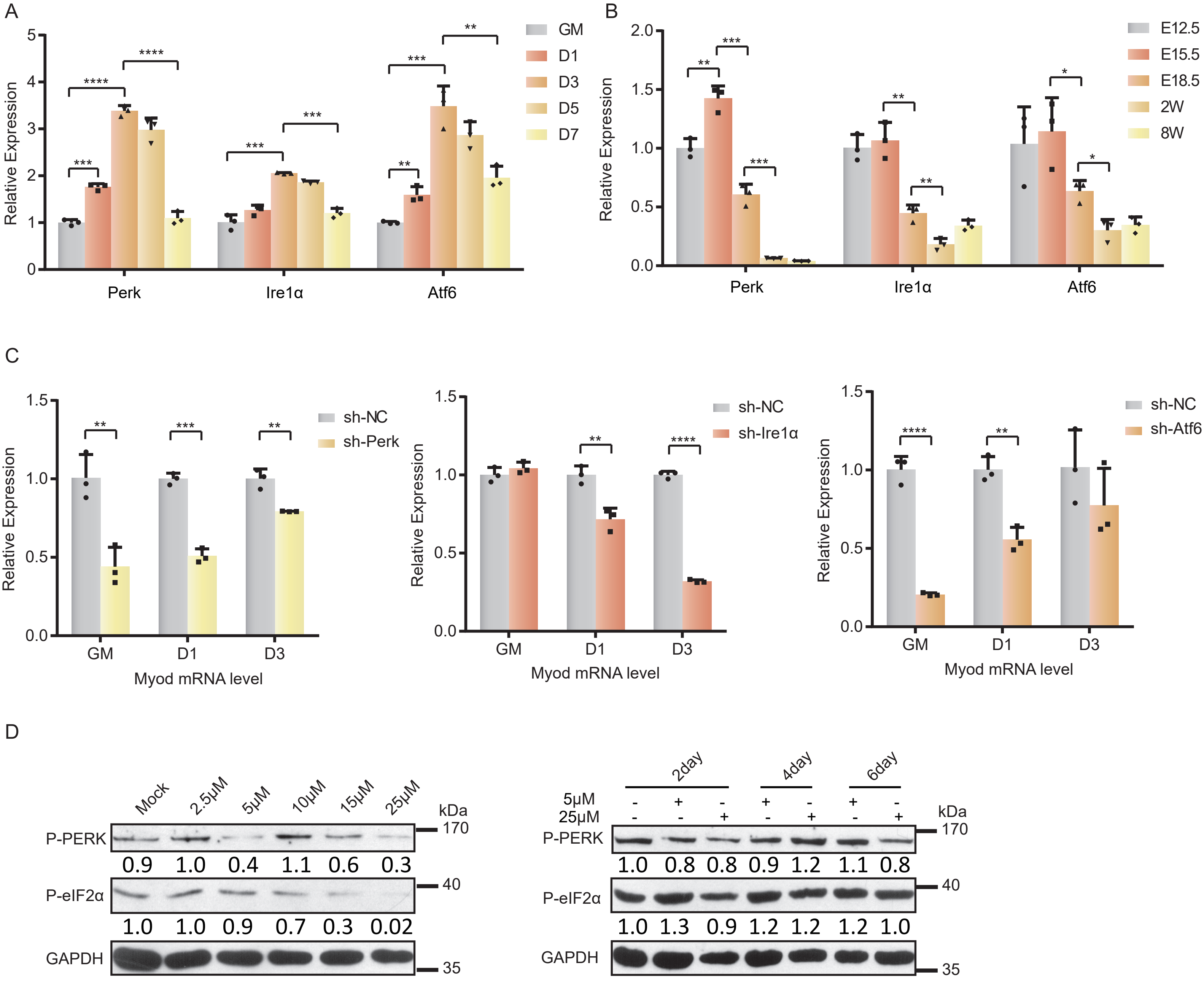
A
Relative Expression
Perk
Ire1α
Atf6
GM
D1
D3
D5
D7
B
Relative Expression
Perk
Ire1α
Atf6
E12.5
E15.5
E18.5
2W
8W
C
Relative Expression
GM
D1
D3
Myod mRNA level
sh-NC
sh-Perk
sh-Ire1α
sh-Atf6
D
Mock
2.5μM
5μM
10μM
15μM
25μM
kDa
P-PERK
170
0.9 1.0 0.4 1.1 0.6 0.3
P-eIF2α
40
1.0 1.0 0.9 0.7 0.3 0.02
GAPDH
35
2day
4day
6day
5μM
25μM
P-PERK
1.0 0.8 0.8 0.9 1.2 1.1 0.8
P-eIF2α
1.0 1.3 0.9 1.2 1.2 1.2 1.0
GAPDH

Figure S2

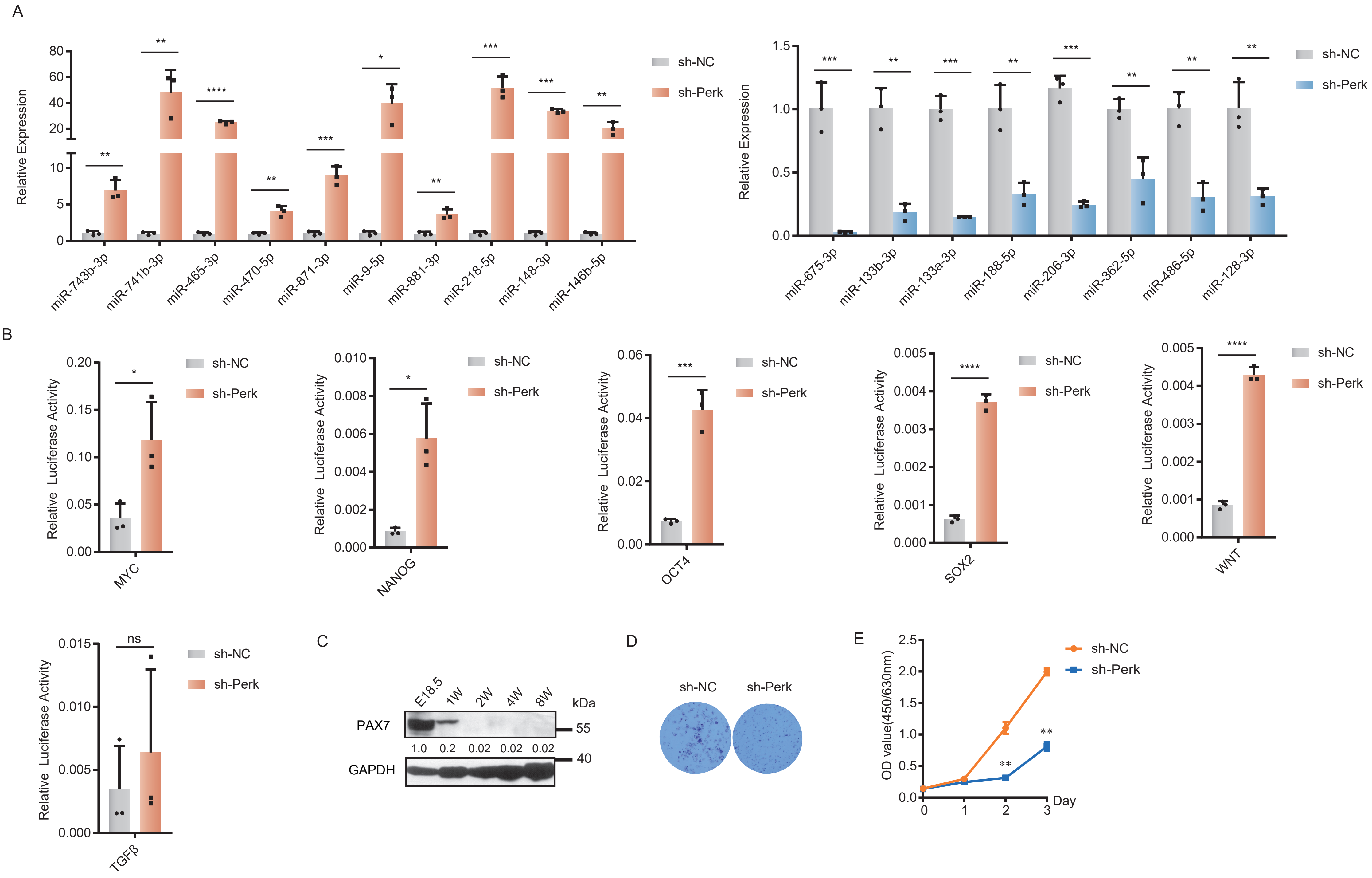
A
Relative Expression
80
60
40
20
10
5
0
**
**
****
**
***
*
**
***
***
**
miR-743b-3p
miR-741b-3p
miR-465-3p
miR-470-5p
miR-871-3p
miR-9-5p
miR-881-3p
miR-218-5p
miR-148-3p
miR-146b-5p
sh-NC
sh-Perk
Relative Expression
1.5
1.0
0.5
0.0
***
**
***
**
***
**
**
**
miR-675-3p
miR-133b-3p
miR-133a-3p
miR-188-5p
miR-206-3p
miR-362-5p
miR-486-5p
miR-128-3p
sh-NC
sh-Perk
B
Relative Luciferase Activity
0.20
0.15
0.10
0.05
0.00
*
MYC
sh-NC
sh-Perk
Relative Luciferase Activity
0.010
0.008
0.006
0.004
0.002
0.000
*
NANOG
sh-NC
sh-Perk
Relative Luciferase Activity
0.06
0.04
0.02
0.00
***
OCT4
sh-NC
sh-Perk
Relative Luciferase Activity
0.005
0.004
0.003
0.002
0.001
0.000
****
SOX2
sh-NC
sh-Perk
Relative Luciferase Activity
0.005
0.004
0.003
0.002
0.001
0.000
****
WNT
sh-NC
sh-Perk
Relative Luciferase Activity
0.015
0.010
0.005
0.000
ns
TGFβ
sh-NC
sh-Perk
C
E18.5
1W
2W
4W
8W
kDa
PAX7
55
1.0
0.2
0.02
0.02
0.02
40
GAPDH
D
sh-NC
sh-Perk
E
OD value(450/630nm)
2.5
2.0
1.5
1.0
0.5
0.0
0
1
2
3
Day
**
**
sh-NC
sh-Perk

Figure S3

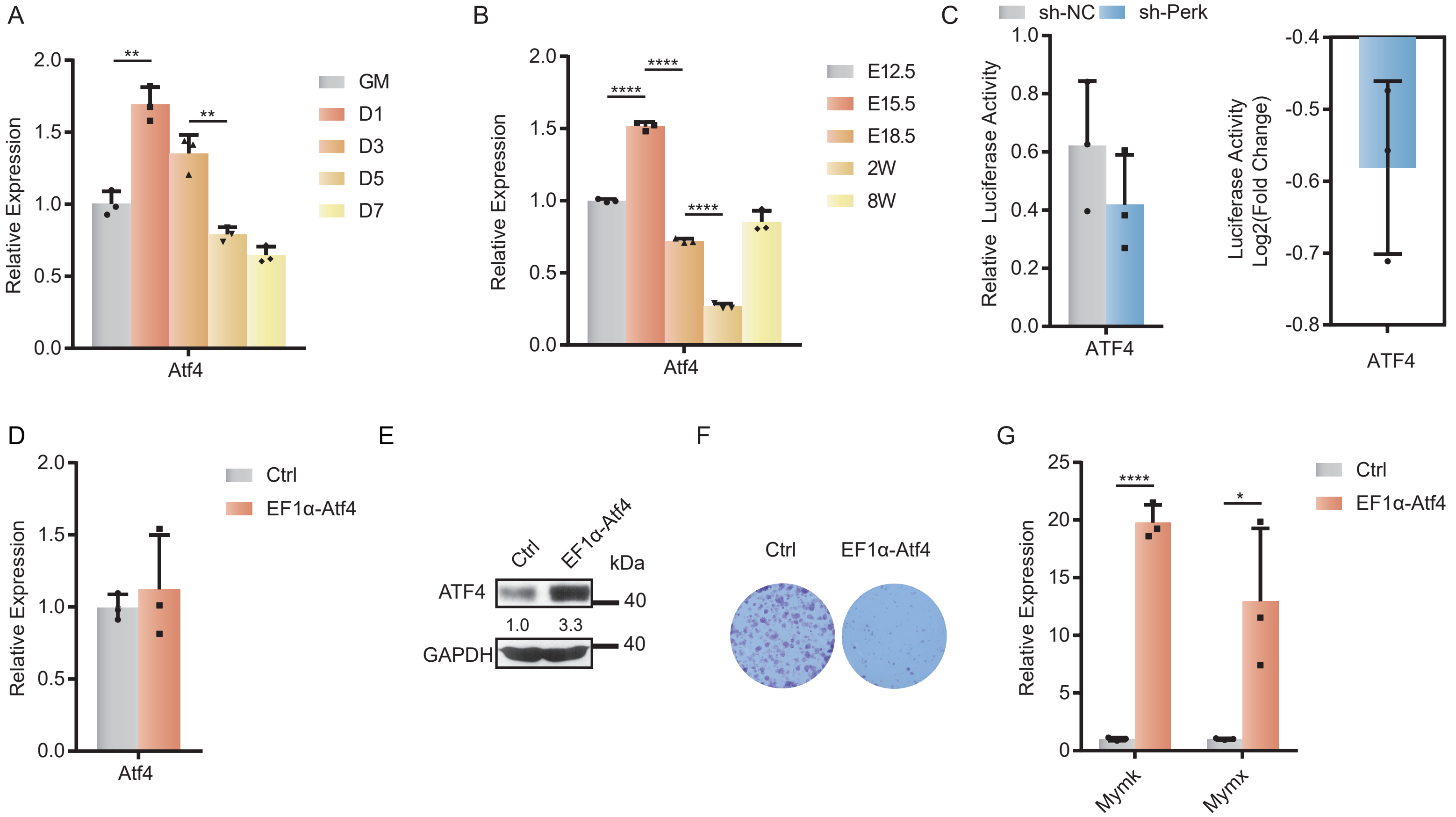
A
Relative Expression
Atf4
GM
D1
D3
D5
D7
**
B
Relative Expression
Atf4
E12.5
E15.5
E18.5
2W
8W
****
C
sh-NC
sh-Perk
Relative Luciferase Activity
ATF4
Luciferase Activity Log2(Fold Change)
D
Relative Expression
Atf4
Ctrl
EF1α-Atf4
E
Ctrl
EF1α-Atf4
kDa
ATF4
40
1.0
3.3
GAPDH
F
G
Relative Expression
Mymk
Mymx
*

Figure S4

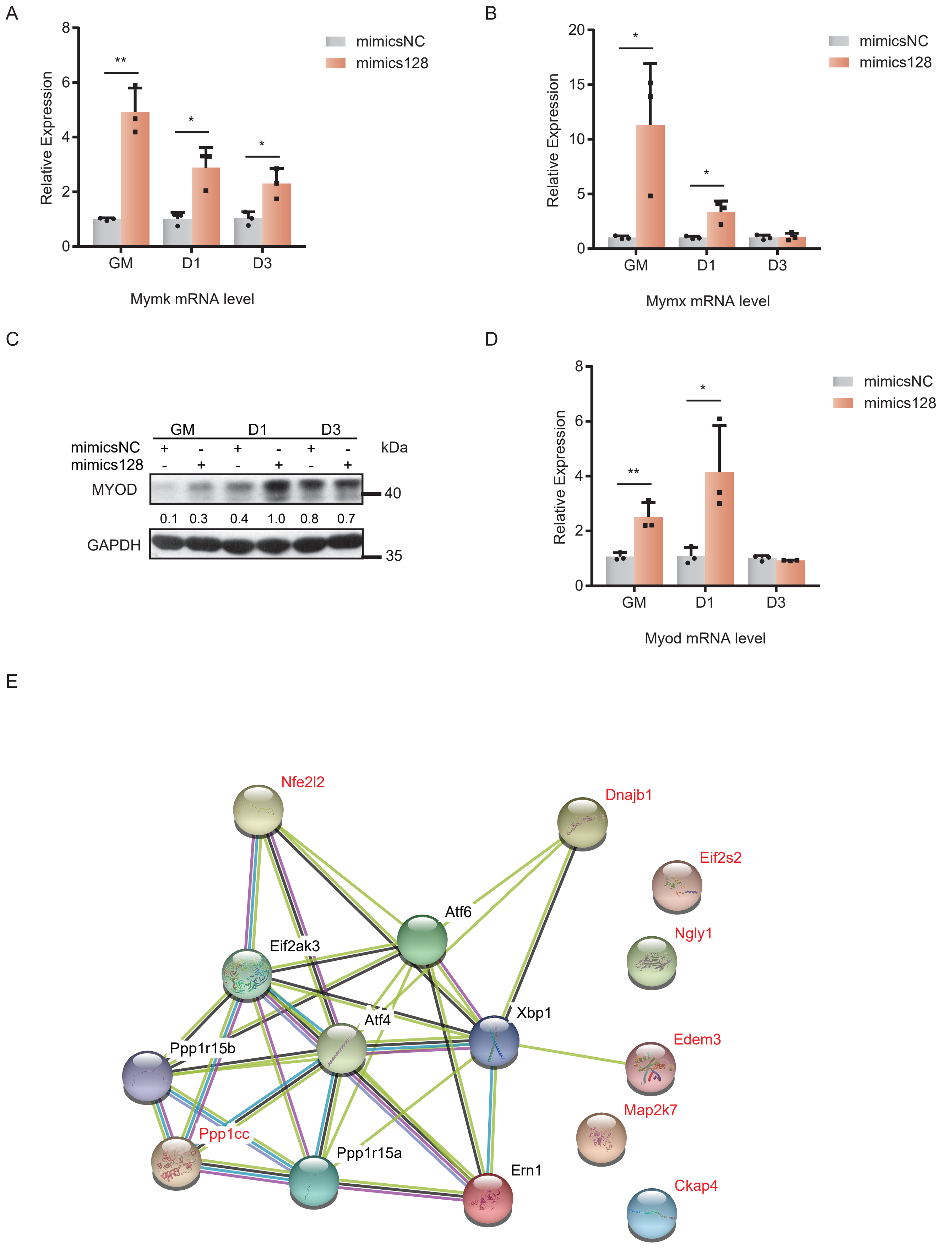
A
Relative Expression
8
6
4
2
0
**
*
*
GM
D1
D3
mimicsNC
mimics128
Mymk mRNA level
B
Relative Expression
20
15
10
5
0
*
*
GM
D1
D3
mimicsNC
mimics128
Mymx mRNA level
C
GM
D1
D3
mimicsNC
mimics128
kDa
MYOD
40
0.1 0.3 0.4 1.0 0.8 0.7
GAPDH
35
D
Relative Expression
8
6
4
2
0
*
**
GM
D1
D3
mimicsNC
mimics128
Myod mRNA level
E
Nfe2l2
Dnajb1
Eif2s2
Atf6
Ngly1
Eif2ak3
Xbp1
Atf4
Edem3
Ppp1r15b
Map2k7
Ppp1cc
Ppp1r15a
Ern1
Ckap4

Figure S5

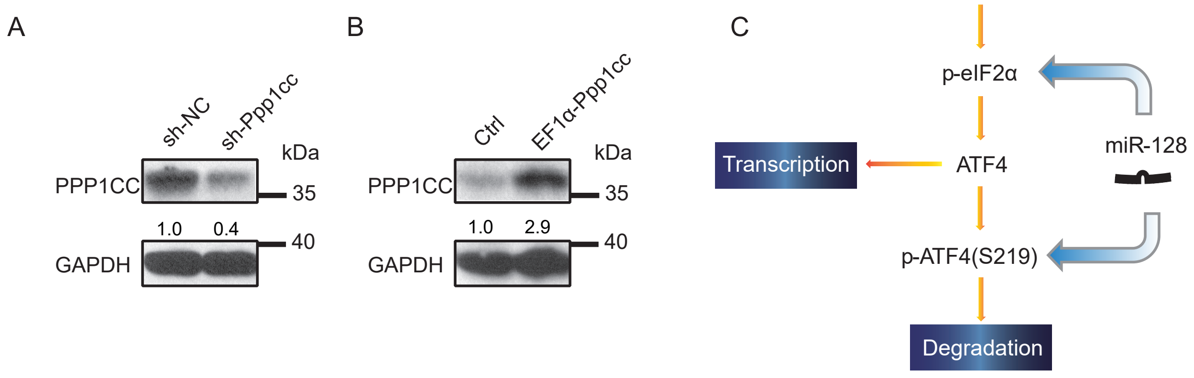
A
sh-NC
sh-Ppp1cc
kDa
PPP1CC
35
1.0
0.4
40
GAPDH
B
Ctrl
EF1α-Ppp1cc
kDa
PPP1CC
35
1.0
2.9
40
GAPDH
C
PERK
p-eIF2α
Transcription
ATF4
miR-128
p-ATF4(S219)
Degradation

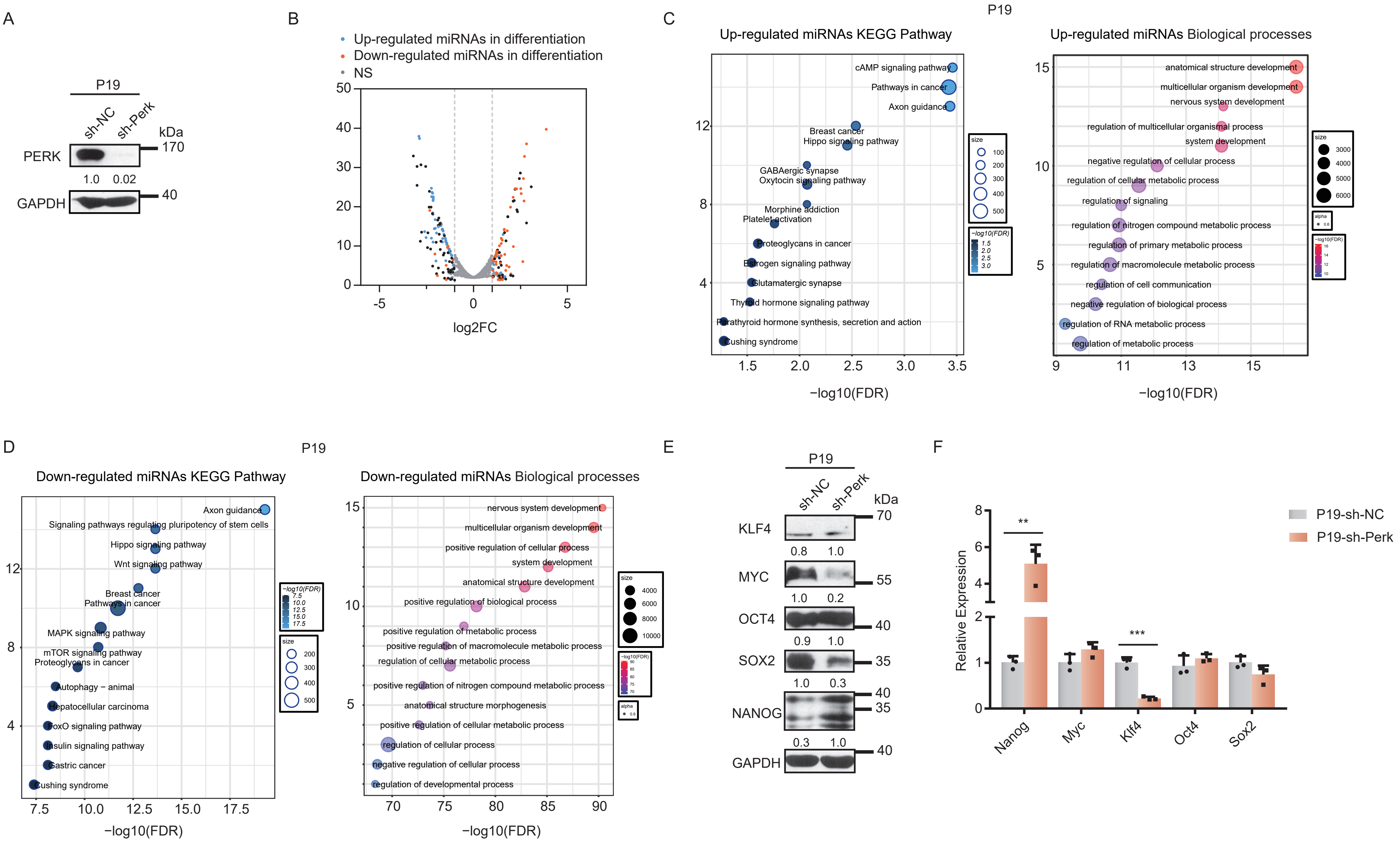
A
P19
sh-NC
sh-Perk
kDa
PERK
170
1.0
0.02
GAPDH
40
B
Up-regulated miRNAs in differentiation
Down-regulated miRNAs in differentiation
NS
log2FC
C
P19
Up-regulated miRNAs KEGG Pathway
cAMP signaling pathway
Pathways in cancer
Axon guidance
Breast cancer
Hippo signaling pathway
GABAergic synapse
Oxytocin signaling pathway
Morphine addiction
Platelet activation
Proteoglycans in cancer
Estrogen signaling pathway
Glutamatergic synapse
Thyroid hormone signaling pathway
Parathyroid hormone synthesis, secretion and action
Cushing syndrome
−log10(FDR)
Up-regulated miRNAs Biological processes
anatomical structure development
multicellular organism development
nervous system development
regulation of multicellular organismal process
system development
negative regulation of cellular process
regulation of cellular metabolic process
regulation of signaling
regulation of nitrogen compound metabolic process
regulation of primary metabolic process
regulation of macromolecule metabolic process
regulation of cell communication
negative regulation of biological process
regulation of RNA metabolic process
regulation of metabolic process
−log10(FDR)
D
P19
Down-regulated miRNAs KEGG Pathway
Axon guidance
Signaling pathways regulating pluripotency of stem cells
Hippo signaling pathway
Wnt signaling pathway
Breast cancer
Pathways in cancer
MAPK signaling pathway
mTOR signaling pathway
Proteoglycans in cancer
Autophagy − animal
Hepatocellular carcinoma
FoxO signaling pathway
Insulin signaling pathway
Gastric cancer
Cushing syndrome
−log10(FDR)
Down-regulated miRNAs Biological processes
nervous system development
multicellular organism development
positive regulation of cellular process
system development
anatomical structure development
positive regulation of biological process
positive regulation of metabolic process
positive regulation of macromolecule metabolic process
regulation of cellular metabolic process
positive regulation of nitrogen compound metabolic process
anatomical structure morphogenesis
positive regulation of cellular metabolic process
regulation of cellular process
negative regulation of cellular process
regulation of developmental process
−log10(FDR)
E
P19
sh-NC
sh-Perk
kDa
KLF4
70
0.8
1.0
MYC
55
1.0
0.2
OCT4
40
0.9
1.0
SOX2
35
1.0
0.3
NANOG
40
35
0.3
1.0
GAPDH
40
F
P19-sh-NC
P19-sh-Perk
Relative Expression
**
***
Nanog
Myc
Klf4
Oct4
Sox2
